# Predictive value of an unsupervised web-based assessment of the neuropsychological function

**DOI:** 10.1038/s41598-025-85614-x

**Published:** 2025-01-10

**Authors:** Michelle Werrmann, Yesim Gür-Tok, Michael Eid, Michael Niedeggen

**Affiliations:** 1 Division of Experimental Psychology and Neuropsychology, Department of Education and Psychology, Freie Universität Berlin, Habelschwerdter Allee 45, 14195 Berlin, Germany; 2 Division of Methods and Evaluation, Department of Education and Psychology, Freie Universität Berlin, Berlin, Germany

**Keywords:** Neuropsychological diagnostic, Web-based assessment, Predictive value, Memory, Attention, Human behaviour, Diagnosis, Neurological disorders

## Abstract

Neuropsychological assessment has to consider the subjective and objective functional deficits of help-seeking individuals in several cognitive domains. Due to time constraints in clinical practice, several web-based approaches have been developed. The current study examined whether functional deficits in the mnestic and attentive domain can be predicted based on an unsupervised self-administered online assessment neuropsychological online screening (NOS): This screening includes self-reports and psychometric memory tests (face-name association, visual short-term memory). Data of help-seeking individuals (n = 213, mean age: 48.2 years) running an extensive in-person assessment were analyzed. A functional deficit in at least one cognitive domain was detected in 48 individuals. This classification was supported by the preceding online screening (sensitivity = 0.75, specificity = 0.80), and a linear regression model identified two significant predictors (free recall performance, form discrimination in visual short-term memory). The predictive quality was enhanced for individuals with selective deficits in the mnestic domain (n = 23: sensitivity = 0.78 and specificity = 0.78) as compared to the attentive domain (n = 25: sensitivity = 0.68 and specificity = 0.75). Our results show that a neuropsychological in-person assessment cannot be replaced by an unsupervised self-administered online test. However, a specific pattern of results in the online test might indicate the necessity of an extensive assessment in help-seeking individuals.

## Introduction

Neuropsychological diagnostics is an essential procedure in different domains of the health system: In neurology, for example, disorders are often associated with cognitive deficits^1^. The neuropsychological examination provides a cognitive profile covering different domains (e.g. memory, attention, or executive functions) which can be related to problems observed in daily living. A suspected neurological disorder can be validated if the patients’ neuropsychological profile matches the characteristic pattern. For example, an early stage of Alzheimer’s disease can be characterized by problems in episodic memory while vascular dementia is more closely related to deficits in semantic memory, attention and executive function^2^. Correspondingly, the choice of the psychometric instruments to be used in the neuropsychological assessment is defined by the suspected neurological disorder.

However, neuropsychological diagnosis is not limited to the confirmation of neurological disorders: Besides of practical issues, such as the assessment of driving abilities^3^, it is also indicated if—otherwise healthy—individuals report a cognitive decline. Depending on several factors, such as personal health concerns, family history of neurodegenerative diseases and psycho-affective state, these individuals might be motivated to show a problem-focused help-seeking behavior^4^. Given the high prevalence of subjective cognitive impairments (SCI)^5,6^, the group of help-seeking individuals is correspondingly expanding. In contrast to the aforementioned group of neurological patients, the choice of psychometric test instruments depends on the functional deficits subjectively reported by the help-seeking individual.

Depending on the outcome of the diagnostic procedure, different interventions might be indicated: If subjective cognitive impairments in a help-seeking individual are confirmed by the result of the neuropsychological assessment, the choice of the intervention depend on the expression of the functional deficit: Individuals with a mild deficit in a single domain might profit from a self-administered training program^7^, whereas severe deficits call for a neurological examination and treatment.

Despite of its importance in the health service, the capacity for a neuropsychological examination is often limited. Temporal restrictions lead to the use of screening procedures (e.g. MMSE) which are limited in their sensitivity. For example, early stages of a neurodegenerative disease cannot be detected reliably if the assessment is based on screening instruments^8^. According to the guidelines^9^, the standard procedure foresees the examination of different domains, such as attention, memory, executive functioning, language and visuo-constructive abilities.

These restrictions affected the development of web-based approaches^10^: Here, computer-based versions of neuropsychological instruments are made available on a web platform, and become more-easily accessible and time saving for specialists—and clients. In a supervised version, these apps can be performed via videoconferencing. More challenging is the unsupervised use. A self-administered online assessment has the advantage to save personal and time resources, and allows to monitor the status of are large number of help-seeking individuals^10^. However,—as compared to in-person testing—the reliability of the results is questioned by several factors: First, access to self-administered assessment is limited. Its use requires access to a computer/tablet with internet access. More importantly, running the procedure unsupervised requires experience with internet application. Both requirements may not apply to some older individuals^11^. Second, the web-based application also has to fulfill psychometric criteria, such as validity and reliability. In case of an unsupervised application, usability has to be added: the procedure should be brief and each task must be comprehensibly explained. Finally, the performance of an individual has to be related to standard normative values^10^. Third, self-administered online assessment are usually restricted with respect to the functional domains tested. Productive language and visuo-constructive functions are usually not considered^12^. Also, the measurement of response times is often unreliable due to different technical capabilities of home devices used by help-seekers^13^.

Although numerous unsupervised assessment tools are available^12^, only few applications fulfill the criteria defined above. Moreover, the application of the tools might be combined with two further drawbacks: First, the app might be tailor-made for the diagnostics of specific neurological disorders. This applies to the SAGE^14^ or BrainCheck^15^, which are focusing on symptoms of early dementia or traumatic brain injury. Second, subjective cognitive problems or the psycho-affective state might not be considered when using the web-based instruments. These restrictions complicate the identification of help-seeking individuals which might be defined as SCI patients. Furthermore, it is not clear whether the test procedure chosen adequately represents the functional deficits experienced in daily living. Therefore, an application that is more sensitive and focusses on a wider range of information is necessary.

To meet these additional requirements, the Neuropsychological Online Screening (NOS) has been developed^16^. The NOS is an unsupervised web-based screening including a combination of standardized self-reports and standardized psychometric tests. The self-reports focus on the expression of subjective cognitive impairment (CPI)^17^ and psycho-affective state (GDS)^18^. The standardized psychometric tests include a test for visual working memory which requires the binding of visual features. The setup of the test is suitable for its implication in a web-based app. More importantly, the cognitive process of binding has shown a high sensitivity in the early detection of neurodegenerative diseases^19,20^. Additionally, it includes a face-name association test (FNAT). A previous study already indicated that the FNAT allows a valid and reliable estimation of the major indices of learning, retrieval and recognition processes^16^. In contrast to other verbal learning-and-memory tests, such as the VLMT^21^, the test is more suitable for a web-based adaptation. The reliability and validity of the tests included in the NOS has been tested, and normative values for different age groups are available^16,17,22^. Finally, the usability of the NOS has been confirmed: Healthy elderly individuals tested in groups (see methods: normative sample) did not require support when running the web-based application: They were able to understand the instructions and navigate within the program independently. Furthermore, the broad majority of help-seeking individuals invited to run the NOS were able to run the procedure without problems at home. In a qualitative post-hoc interview, none of the help-seeking individuals complained about the clarity of the instructions, and missing NOS data in this sample were exclusively attributed to technical problems (e.g., internet connection, browser issues).

This paper will focus on the predictive value of the self-administered online instrument NOS. The NOS was not designed to replace the in-person examination. Rather, the unsupervised self-assessment should guide the upcoming neuropsychological examination in a memory clinic: The standardized self-reports provide an idea which cognitive domains are—subjectively—mostly affected^17^, and should therefore be considered. The performance in the FNAT and in the binding task should provide first insight whether the mnestic problems have to be considered^16^, or if the assessment can focus on attentional functions not covered in the NOS.

Similar questions had been raised in previous studies on web-based instruments. In most cases, these studies focused on the convergent validity of the unsupervised at-home testing and a supervised testing. This validity was provided for computerized versions of the Stroop and reasoning tasks^23^, as well as for subtests of the Cambridge Neuropsychological Tests Automated Battery (CANTAB) like pair association learning or spatial working memory^13^. Other studies^24,25^ used composite scores, and reported high correlations between the performance in the web-based test (Survey for Memory, Attention, and Reaction Time (SMART) and Cogstate Brief Battery (CBB), respectively) and the cognitive status as estimated by gold-standards.

The current study did not use a correlative approach, but focuses on individual’s test performance: As mentioned above, the result of the neuropsychological examination provides information on the necessity of an intervention. Therefore, we asked whether—subjective and/or objective—deficits in the self-administered online instrument NOS allows a prediction on whether the lab-based assessment will signal that an intervention is necessary. In contrast to the correlation of test scores, the congruency in the classification of an individual’s test performance appears to meet the requirements in daily practice.

In the first step, we analyzed if deficits in the NOS allow a prediction on whether the necessity of an intervention is signaled in the in-person examination—independently of the functional domain. Possible predictors are (1) deficits in the binding task, and (2) recognition problems: Previous findings signal that performance should be at ceiling, and that deviances are most likely to reflect a neurological deficit^26,27^.

In the second step, we differentiated between the functional domain (memory, attention, and executive function) in which an intervention is indicated. We hypothesize that a domain specific prediction is possible. Previous results indicated that the level of subjective complaints is more closely related to memory as compared to attentional deficits^16,28^. Moreover, the functional tests embedded in the NOS are closely related to the mnestic tests. Therefore, we assumed that the NOS is more sensitive in the prediction of functional memory deficits.

## Methods

### Participants

This study was conducted in accordance with relevant guidelines and regulations and the study protocol was approved by the ethics committee of FU Berlin (No. 011/2022). All participants signed informed consent. 213 help-seekers were recruited from the Neuropsychological Consultation Hour of FU Berlin. The Neuropsychological Consultation Hour is a memory clinic at FU Berlin on campus that is open to adult help-seekers with cognitive complaints from the community. Some help-seekers are referred to the Consultation Hour by their attending neurologist or GP.

Three participants were excluded due to a deviance in the performance in the color-condition of the binding task: Normative data indicated a ceiling effect in this condition, and a corresponding deviance (near-chance performance) is likely to reflect deficits in understanding the unsupervised task. The mean age of the remaining 131 female and 82 male participants was 48.22 years (SD = 15.52 years) ranging from 18 to 85 years of age. Regarding the education level, 137 participants reported completing the German Abitur or Fachhochschulreife (12 years of school or more), while 76 participants reported less than 12 years of schooling. 38 people reported suffering from a neurological or psychiatric condition. We did not remove these participants from the sample as removing them did not alter the results reported.

### Stimuli and procedure

Participants concluded the NOS (CPI, GDS, FNAT, STMB test) unsupervised at home via a link to the online platform Pavlovia (https://run.pavlovia.org/nelep92/kos, coding is available via request). Approximately one week later they completed an in-person testing at the neuropsychological consultation hour of FU Berlin. Here, the selection of the test procedure was determined by the profile of the individuals’ cognitive complaints.

The NOS comprises two questionnaires based on self-reports: The Complainer Profile Identification (CPI)^17^ is a questionnaire consisting of 17 items to measure SCI. The items of the CPI were designed to relate to daily functions and frequently reported subjective complaints for increased ecological validity. For example, one item is “If someone tells me something, I have to write it down immediately, so that I do not forget about it.”. The participants are asked to rate the item according to the frequency they experience that problem in their daily life on a 5-point rating scale (from 1 = never to 5 = very often). As a variable, the mean level of cognitive complaints for each participant is extracted and compared to normative data of healthy controls. The Geriatric Depression Scale (GDS)^18^ is a questionnaire measuring depressive mood. Importantly, for this study we used GDS as a measure for depressive mood only as it has been done in previous studies^29^, not as a measure for a clinical diagnosis. The GDS is designed for primarily use with elderly patients and was specifically chosen for the main target group of the neuropsychological consultation hour. Items such as “Are you in a good mood most of the time?”, need to be answered with yes or no. A sum score of answers affirming depressive mood is extracted for each participant.

The two psychometric tests included are focusing on the cognitive performance level. Because of the unsupervised test mode and the differences in technical facilities, response modes were kept simple and did not imply a speeded response.

**Fig. 1:**
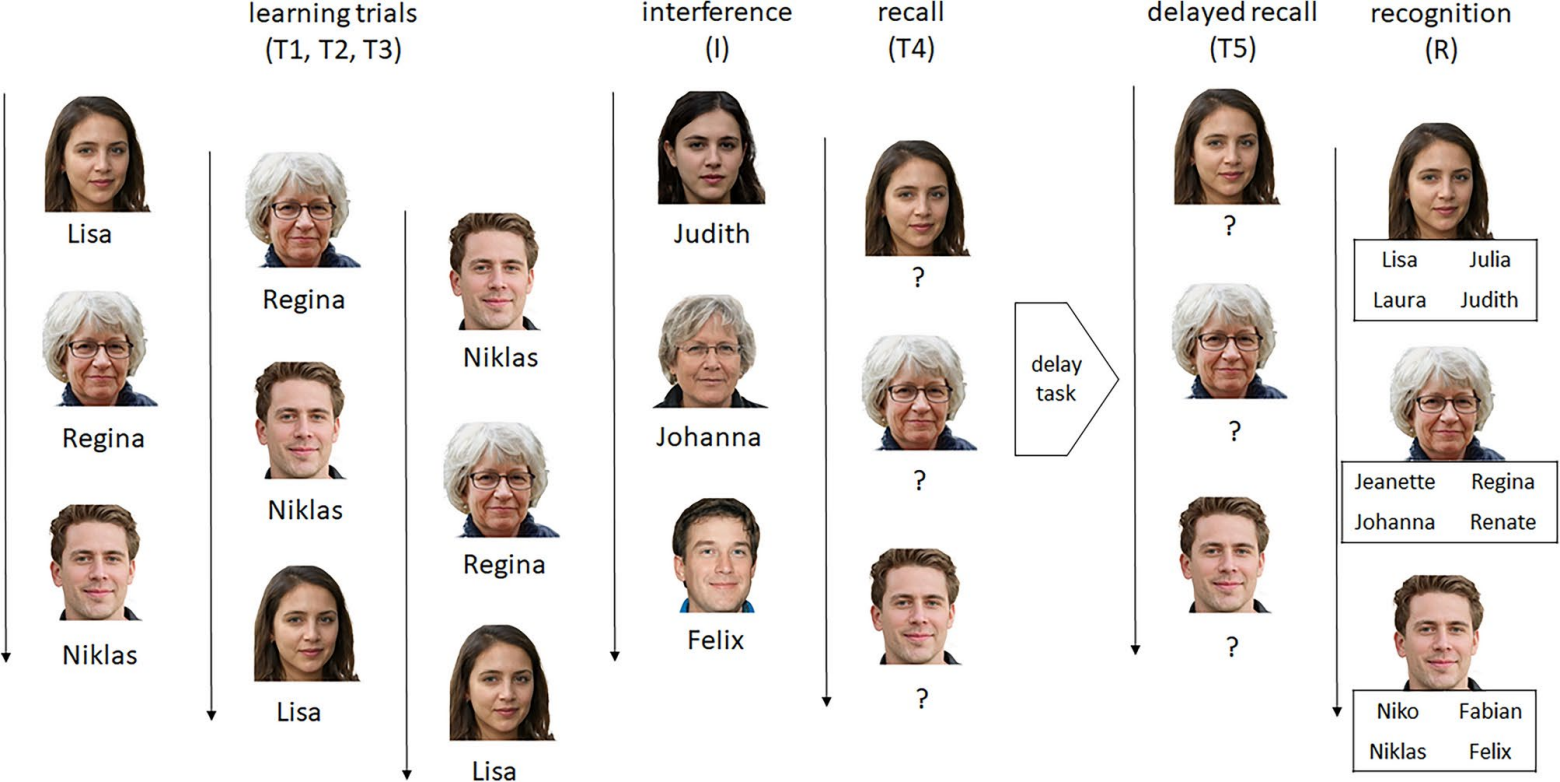
Sequence of FNAT trials. In the learning trials 12 faces are shown consecutively with an associated name underneath. After each learning trial an immediate recall (T1, T2, T3) is asked of the participant. After three learning trials, an interference task (I) is commenced in the same manner with 12 matched faces. Next, the participants are asked to recall the original face-name associations (T4) without additional presentation. After a delay task, a delayed recall trial (T5) is undertaken. Lastly, in a recognition trial (R), the participants can choose the correct name from four alternatives. Please note that all faces are computer-generated.

The Face-name association test (FNAT) is a performance test measuring associative learning and memory performance. Two sets of 12 computer-generated faces are presented with an associated name underneath each of them as can be seen in Fig. 1. Four independent raters rated the faces as realistic and they depict people in a broad age range, both male and female. Both sets of faces were matched for sex, age and global characteristics. Names were chosen based on high frequency of those names in the German general population matching the perceived age of the respective face. Names and faces were chosen to resemble people that would be most familiar with the expected older target group of the neuropsychological consultation hour, resulting in an age-diverse but otherwise rather homogenous group (Caucasian, no tattoos or body modification). Before the first trial (T1) a practice trial is recommenced. We extracted a sum score for the learning phase (T1 + T2 + T3), a delayed recall score (T5) and a recognition score (R) for each participant.

In the learning trials (T1, T2, T3), participants are shown the 12 faces of the first set sequentially with an associated name underneath. For an example, please refer to Fig. 1. Each face is presented for 2 s. The participant is asked to memorize the faces with the associated names. Then the faces are shown again in a randomized order and the participant is to enter the first letter of the memorized name or “X” when they do not remember. This initial learning phase is repeated three times. Then the 12 matched faces and names of the interference trial (I) are presented and tested in the same manner. Afterwards, the initial set of faces and names are tested again in free recall (T4), without an additional learning phase. After a distractor task (approximately 15–20 min), a delayed recall (T5) is administered. Lastly, in a recognition trial (R) each of the initial 12 faces are shown with four possible names and the participant is asked to select the correct one. To ensure that the participants remember the full name and not just the first letter, here, two out of the four names start with the same letter.

**Fig. 2:**
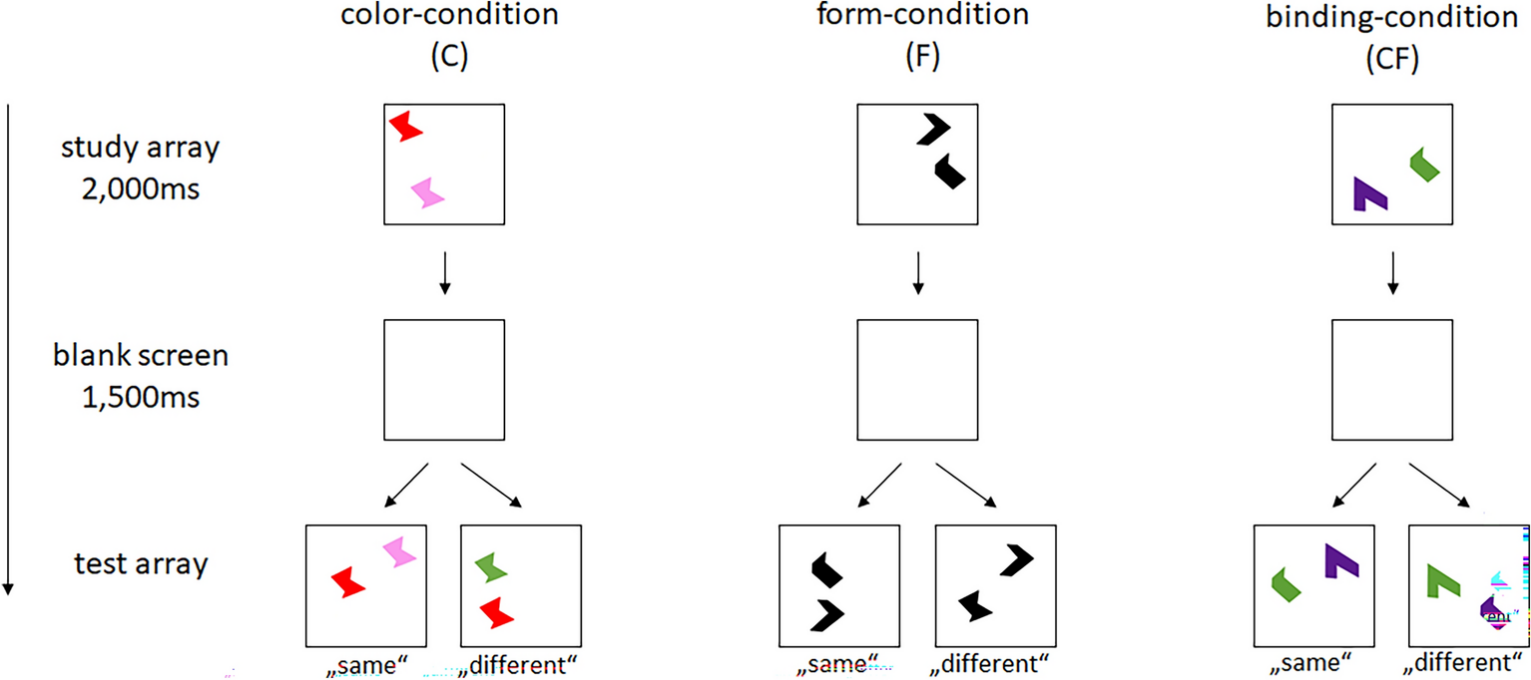
The short-term memory binding test (STMB Test) has three conditions. The study array is shown for 2000 ms. After a 1500 ms blank screen, the participants are asked to indicate whether the test array is the same as the study array regarding the condition task.

The short-term memory binding test (STMB Test) is a performance test measuring visual short-term memory and binding performance. This test has been established as diagnostic tool relating to neurodegenerative disease^20,29,30^. In the study array, as can be seen in Fig. 2, two unfamiliar shapes are shown in different colors. Depending on the condition (color, form, binding), the participants are asked after a short waiting period whether the colors, forms or both in the test array are the same or different as in the study array. The discrimination index A′^31^, based on the ratio of hits to false alarms, was extracted for each participant for each condition.

The general procedure is depicted in Fig. 2. In the color-condition, two shapes in two different colors are presented in the study array. After a short break of 1500 ms, a test array with the two shapes is shown and the participant is asked to indicate, by pressing a button on the keyboard or screen, whether the colors are identical to the study array. In the form-condition, the two unfamiliar shapes are presented in the same color during the study array and the participant has to decide whether the test array shows the same shapes. In the binding-condition, two shapes are shown in two colors during the study array. In the test array the same shapes and colors are presented, however, the participant has to indicate whether the shapes are matched with the same color as before. Each condition is preceded by a practice trial where feedback is given on the correct or incorrect answers. The practice trials can be repeated if the participant chooses to do so. Each condition consists of 64 trials (32 “same” trials, 32 “different” trials). The color-condition served as a control condition to check for task understanding.

### Examination procedure

**Fig. 3:**
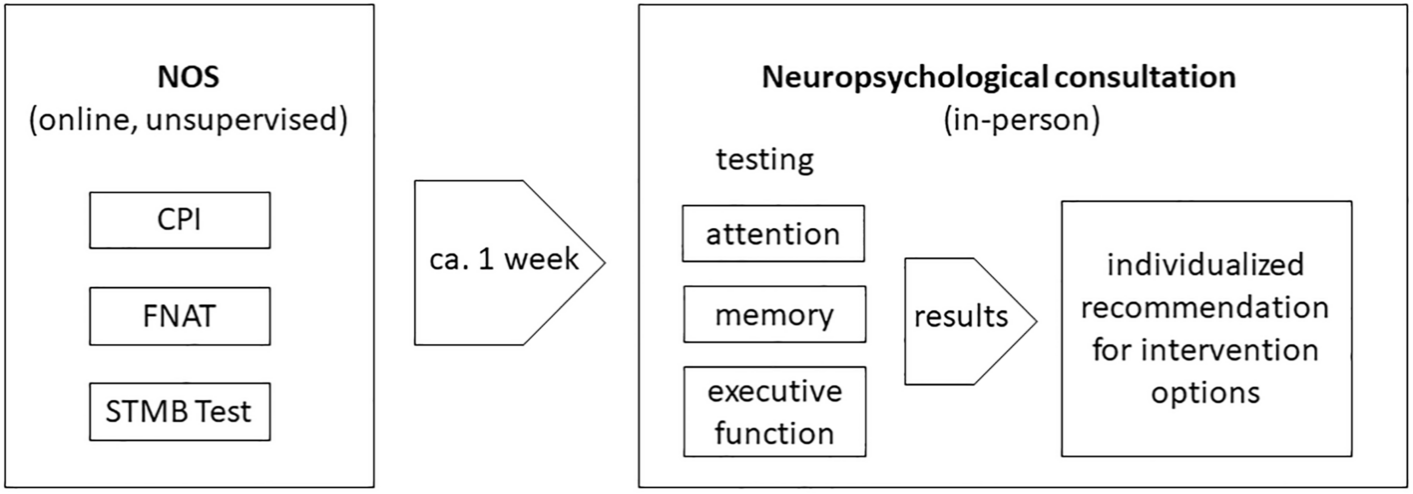
Timeline of the experimental procedure. Participants concluded the NOS (including CPI, FNAT, and STMB Test) unsupervised and online at home approximately 1 week before the in-person testing in the neuropsychological consultation hour. Here, performance in attention, memory, and executive function were measured and, based on the results, individualized recommendations for intervention options were given.

Each help-seeking individual requesting an appointment at the neuropsychological consultation hour at the FU Berlin was also invited to complete the NOS in advance. Via email, the help-seeker was sent a link to the online platform. Individuals were instructed to complete the NOS approximately one week before the appointment. At the date of the appointment at the neuropsychological consultation hour, a semi-structured interview—based on the CPI results—was conducted to identify and/or confirm subjective cognitive problems. Based on the outcome, appropriate psychometric tests were selected. In all help-seekers, the three cognitive domains ‘memory’, ‘attention’, and ‘executive functions’ were covered. Following the completion of the tests, the results were analyzed, and feedback was provided to the help-seeker. Help-seekers were also asked whether they experienced problems with the NOS (e.g. clarity of instructions). The timeline of this process is detailed in Fig. 3.

As mentioned above, psychometric tests for the in-person examination were selected on a case-by-case basis depending on the concerns and general performance level of the help-seeker. In the majority of individuals, subtests of the TAP^32^ were applied covering different attentional domains, as well as executive functions: In a selective attention task, deviances in a series of auditory tones have to be detected (tone repetitions). The median of the reaction time reflects processing speed, the standard deviation the stability, and the number of misses the accuracy. Correspondingly, in the shared attention task, a series of auditory tones and visual stimuli has to be monitored, and predefined targets required a speeded response. Here, the estimation of accuracy is based on false positive reactions and misses. As for the mnestic domain, learning rate, free recall, and recognition (see above) were examined in an established verbal learning-and-memory test. Depending on the age of the participant, either the VLMT (Verbaler Lern-und Merkfähigkeitstest)^21^ in younger (< 50) individuals or the CERAD (Consortium to Establish a Registry for Alzheimer’s Disease)^33^ was applied. Additionally, the majority of participants concluded the digit span test to estimate the working memory capacity^34^. The use of further psychometric tests depended on the profile of subjective cognitive complaints.

### Statistical analysis

Following our experimental rationale, we dichotomized the outcome of the test results. This procedure follows the daily clinical routine, and the use of cut-off values to decide on the impact of a test result.

The NOS results, further labeled as predictors, are the GDS score, mean CPI level, FNAT learning rate, FNAT delayed recall, FNAT recognition score. As for the STMB test, the discrimination indices A′(F) of the form-condition and A′(CF) of the binding condition were considered. The color-condition was used as an indicator of result validity (see above) and participants performing near chance were excluded. Furthermore, the analysis considered the individuals age and education.

The computation of the cut-off points for the NOS relied on the performance level of a normative population. Normative data were based on the examination a large sample of healthy adults (n = 406, age between 18 and 86 years). The majority of data sets were collected under controlled conditions at public scientific annual events taking place at the FU Berlin open to the general public. Please note, that only the NOS data were available in this sample, and that no standard neuropsychological examination took place.

If the performance of an individual was within the 95% range, the dichotomous value ‘0’ was assigned. If the performance was within the lower 5% percentile, a deficit was assumed and the dichotomous value ‘1’ was assigned. For CPI, if the self-rating was within the highest 5% percentile of subjective complaints it was assigned a value of ‘1’ (high SCI) otherwise it was assigned ‘0’ (low SCI). Please note that the use of the conservative cut-off value will increase specificity. As for the self-reports, the GDS was labeled as ‘0’ (no depressive mood) if the score was smaller than 11 points, and ‘1’ (depressive mood) if the score was larger than 10 points^35,36^. We categorized education level in two categories according to the German schooling system: less than 12 years of school (‘0’) and 12 or more years of school (‘1’).

The test scores provided by the in-person examination in the Neuropsychological Consultation Hour, further labeled as dependent variables, were related to the attentive, mnestic and executive function. For each of the domains, we determined whether the test results indicated the necessity of a supervised clinical intervention. The choice of tests in the Neuropsychological Consultation Hour were tailored to the individual’s needs. Therefore, not all participants completed all tests considered in the present study resulting in different sample sizes per cognitive domain.

As for the attention domain, we focused on the performance in the selective attention task, and considered three variables: (1) response time (median), (2) stability of response time (SD) and (3) accuracy (misses). The need of supervised clinical intervention—coded as ‘1’—was indicated if at least two of the performance measures remained at or under the 5% percentile according to the normative data provided in the TAP manual^32^. Otherwise, the corresponding code was set at ‘0’.

As for the mnestic domain, the following z-transformed test values extracted from the CERAD or the VLMT (based on CVLT) were considered: (1) learning performance (learning rate), (2) delayed free recall, and (3) recognition. Again, supervised clinical intervention was defined if at least two of the performance measures remained at or under the 5% percentile, and the corresponding code (1 or 0) was used.

As for the executive domain, we integrated from a divided attention task (1) the number of errors, (2) accuracy (misses), and (3) working memory (digit span). Again, supervised clinical intervention was defined if at least two of the performance measures remained at or under the 5% percentile according to the normative data provided in the TAP^32^ or digit span manual^37^, and the corresponding code (1 or 0) was used. Since our first research question asked whether the NOS signal the necessity of an intervention independently of the domain, we also defined the unspecific variable “intervention”: This variable was set at ‘1’ if at least in one of the domains aforementioned an intervention was indicated by the psychometric test.

For the statistical analyses the programs R Studio^38^ and SPSS^39^were used. In the first analysis, we focused on the variable ‘Intervention’. Data analysis included three steps: In a first step, all predictors were considered in a binomial logistic regression analysis using a general linear model (see supplement). In a second step, all predictors showing a *p*-value of *p* > 0.1 were excluded. With the remaining predictors, a new model was calculated. This step was consecutively repeated until the model only included significant predictors (*p* < 0.05). The reduced models will be reported in the results section. All general assumptions were met. A likelihood ratio *χ*^2^-test was computed to assess model significance. Nagelkerke Index and McFadden’s R^2^ are used as effect size measures. In a final third step, odds ratios were calculated for the individual predictors. This odds ratio reflects the strength of the association.

In the second analysis, the same procedure was applied for the cognitive domain ‘attention’, ‘memory’ and ‘executive function’. The same steps as described above were applied.

## Results

Table 1 summarizes the frequencies of functional deficits based on the results of the neuropsychological in-person assessment in our sample of help-seeking individuals. Please note that the sample size varied depending on whether the execution of the test was possible (see methods).

**Table 1:** Absolute numbers and frequencies of functional deficits requiring intervention based on in-person testing.

Domain	N	No deficit (PR > 5)	Deficit (PR ≤ 5)
Attention	199	N = 174 (87%)	N = 25 (13%)
Memory	188	N = 165 (88%)	N = 23 (12%)
Executive function	197	N = 193 (98%)	N = 4 (2%)
Intervention indicated	182	N = 134 (74%)	N = 48 (26%)

In all domains, a deficit was signaled by a significant deviation in performance in two out of three test scores. Correspondingly, an attentional deficit was observed in 25 out of 199 individuals (12.6%). Memory deficits occurred in 23 out of 165 participants (12.2%). Deficits in the executive function were less frequent and were observed in only 4 out of 197 individuals (2%). In 182 help-seeking individuals, all cognitive domains were tested sufficiently: 26.4% of these individuals showed deficits in at least one cognitive domain. Here, the group of participants with and without a marked deficit did not statistically differ in age, education and GDS score. The corresponding table is provided as a supplement Table S1.

**Fig. 4:**
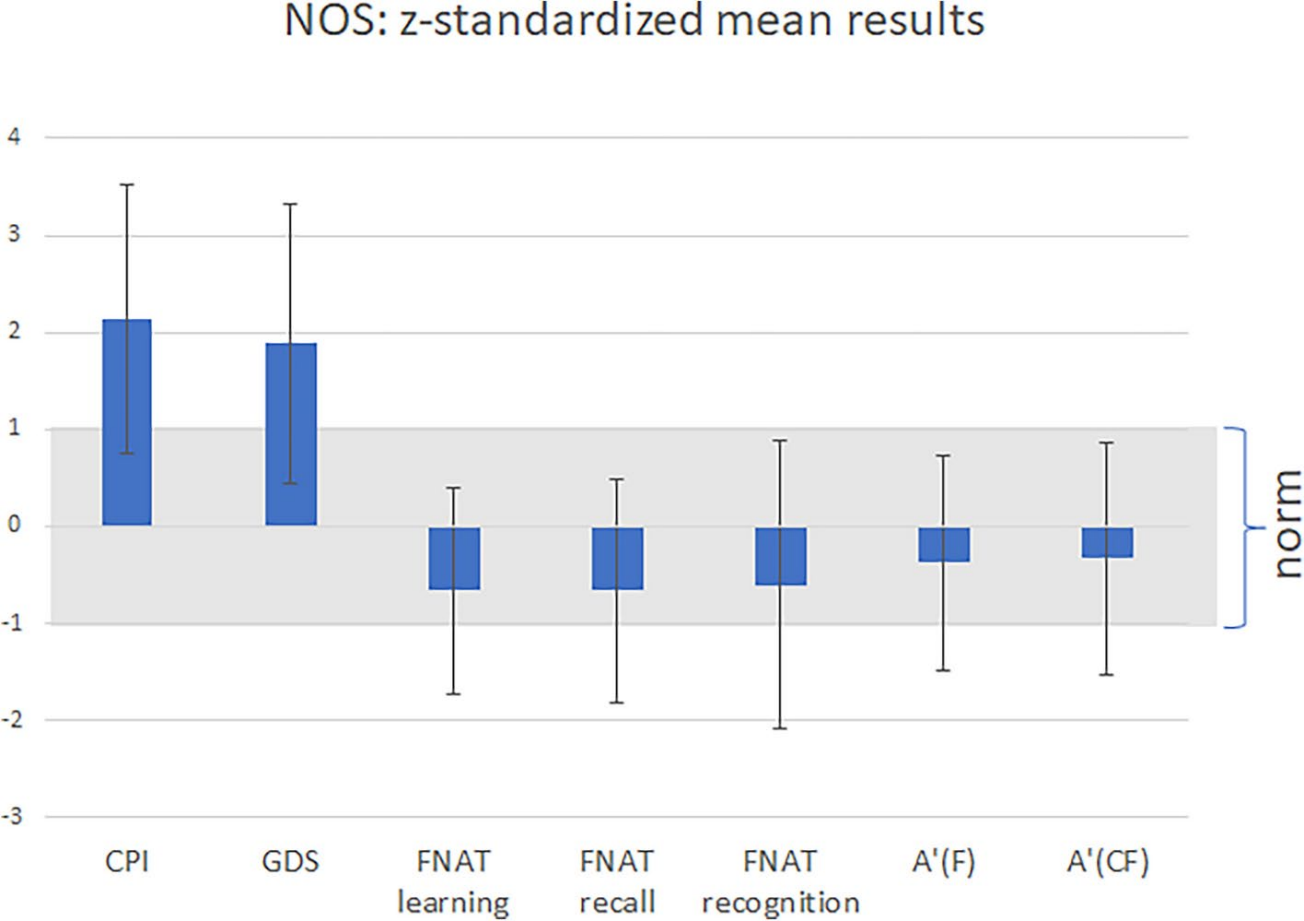
Z-standardized scores of help-seeking individuals (n = 213) in the neuropsychological online screening (NOS). Mean values and standard deviations are depicted. The help-seekers showed an enhanced level of subjective impairment and depressive mood. The performance in the face-name-association-test as well as in the binding task is slightly reduced. Complainer profile identification (CPI), geriatric depression scale (GDS), face-name association test (FNAT), form-condition of short-term memory binding test (A′(F)), binding-condition of short-term memory binding test (A′(CF)).

Figure 4 depicts the level of performance of help-seekers included in this study z-standardized regarding normative values. With respect to the subjective reports, the level of subjective cognitive impairment (mean z = 2.14, SD = 1.39) is increased in help-seeking individuals, as well as the depressive mood (mean z = 1.88, SD = 1.45). The help-seekers displayed on average a descriptively low, z-standardized performance in the FNAT (learning: M = − 0.66, SD = 1.07; recall: M = − 0.66, SD = 1.15; recognition: M = − 0.61, SD = 1.48) and the STMB task (A′(F): M = − 0.37, SD = 1.11; A′(CF): M = − 0.33, SD = 1.20) while still being within the norm.

To estimate in single individuals whether self-reported complaints (CPI, GDS) or deficits in cognitive tests (FNAT, binding) were clearly expressed, a cut-off value was defined (PR < 5). The frequency of deviances separated for the domains of the NOS are provided in Table 2.

**Table 2:** Absolute numbers and frequencies of deviances observed in the Neuropsychological Online Screening (NOS).

Test	N	Normal range (PR > 5)	Deviance (PR ≤ 5)
GDS	213	N = 173 (81%)	N = 40 (19%)
CPI	213	N = 81 (38%)	N = 132 (62%)
FNAT learning	213	N = 171 (80%)	N = 42 (20%)
FNAT delayed recall	213	N = 158 (74%)	N = 55 (26%)
FNAT recognition	213	N = 167 (78%)	N = 46 (22%)
A′(F)	213	N = 189 (89%)	N = 24 (11%)
A′(CF)	213	N = 197 (92%)	N = 16 (8%)

The frequency of highly expressed subjective cognitive problems (62%) was to be expected in help-seeking individuals, and exceed the frequency of self-reported depressive mood (19% within the sample). Deficits in the face-name association test (FNAT) were present in approximately 24% of the sample, whereas problems in the visual short-term memory test occurred less frequently (9%).

### General functional deficit

Following our research questions, the predictability of a functional deficit with a need for intervention—independent of the domain—was analyzed first. In this sample, the sensitivity (correct classification of a neuropsychological deficit on the basis of the NOS result) was 75%: Out of the 48 individuals with a deficit (see Table 1), the NOS already classified 36 correctly. In contrast, the NOS signaled a deficit in 27 individuals out of 134, in which no deviance was observed in the in-person examination. This ratio (here: 20%) will be labeled as ‘false alarm’. The specificity of the model was 0.799.

The results of the regression model—only including the significant predictors—is provided in Table 3. Please note that the complete regression model including all predictors can be found in the supplement. Out of the NOS variables included in the model (see Table 2), the factor ‘FNAT: delayed recall’ and the form discrimination in the short-term memory binding test (A′(F)) served as significant predictors. Based on the odds ratios, the predictive values of the FNAT score was 91.5%—indicating the probability that a deviance in this score, correctly signaled a deficit in the subsequent neuropsychological assessment. The corresponding value of the A′(F) score was 81.5%.

**Table 3:** Logistic regression model: predictors of a necessity for intervention—irrespective of the domain—based on the neuropsychological online screening (NOS) variables and effect size measures for the model.

	B	SE	Wald	df	*p*	Exp(B)	CI Exp(B)
FNAT delayed recall	2.376	0.402	34.941	1	< 0.001***	10.765	[4.896, 23.671]
A′(F)	1.482	0.553	7.180	1	0.007**	4.402	[1.489, 13.015]
Constant	− 2.161	0.290	55.365	1	< 0.001***	0.115	

Chi^2^ (2) = 51.004, *p* < 0.001***

Nagelkerke Index = 0.409.

McFadden’s R^2^ = 0.243.

Significance level: *p* < 0.05*, *p* < 0.01**, *p* < 0.001***

### Attentional deficit

In the next steps of analysis, the corresponding analysis was run for the three neuropsychological domains covered in the in-person assessment.

As for the attention domain, the sensitivity was 68%: 17 out of 25 individuals with attention problems were detected by the NOS. The false alarm rate was 25%, based on 44 misclassifications in 174 individuals without attention problems. The model showed a specificity of 0.747.

The predictors identified in the regression analysis (see Table 4) paralleled those identified in the first analysis: Based on the odds ratios, the predictive values of the FNAT delayed recall score was 83.6%—indicating the probability that an attentional deficit can be observed in the subsequent neuropsychological assessment. The corresponding value of the A′(F) was 81.5%.

**Table 4:** Logistic regression model: predictors of a functional deficit in the attentional domain based on the neuropsychological online screening (NOS) variables and effect size measures for the model.

	B	SE	Wald	df	*p*	Exp(B)	CI Exp(B)
FNAT delayed recall	1.629	0.464	12.328	1	< 0.001***	5.101	[2.054, 12.667]
A′(F)	1.486	0.553	7.223	1	0.007**	4.419	[1.495, 13.060]
Constant	− 2.826	0.349	65.447	1	< 0.001***	.059	

Chi^2^ (2) = 22.704, *p* < 0.001***

Nagelkerke Index = 0.203.

McFadden’s R^2^ = 0.151.

Significance level: *p* < 0.05*, *p* < 0.01**, *p* < 0.001***

### Memory deficit

In the memory domain, the sensitivity was 78%, based on a correct NOS classification of 18 out of 23 individuals with memory problems. The false alarm rate was 22%, and reflected the misclassification of 36 out of 166 individuals without memory problems. The model showed a specificity of 0.782.

The reduced regression model (Table 5) confirmed that the delayed recall in the FNAT serves as a significant predictor. In contrast to the previous analysis, the binding performance in the short-term memory binding task (A′(CF)) defines a second significant predictor. Based on the odds ratios, the predictive values of the FNAT score was 91.1%—indicating the probability that a mnestic deficit can be observed in the subsequent neuropsychological assessment. The corresponding value of the A′(CF) was 83.3%.

**Table 5:** Logistic regression model: Predictors of a functional deficit in the memory domain based on the neuropsychological online screening (NOS) variables and effect size measures for the model.

	B	SE	Wald	df	*p*	Exp(B)	CI Exp(B)
FNAT Delayed recall	2.323	0.554	17.560	1	< 0.001***	10.201	[3.443, 30.229]
A′(CF)	1.605	0.664	5.840	1	0.016*	4.980	[1.354, 18.309]
Constant	− 3.350	0.464	52.213	1	< 0.001***	0.035	

Chi^2^ (2) = 34.051, *p* < 0.001***

Nagelkerke Index = 0.316.

McFadden’s R^2^ = 0.244.

Significance level:* p* < 0.05*, p < 0.01**,* p* < 0.001***

### Executive deficit

A corresponding model was not computed for the executive function: Although the sensitivity was apparently high (100%: 4/4) as contrasted to the false alarm rate (26%: 50/193), a significant regression model could not be determined (see supplement) due to the low probability of a selective deficit in the executive function.

### Overlap between domain-specific deficits

The similarities between the regression model for attentional and mnestic deficits might elicit the question of on overlap between these functional domains. Therefore, we have to identify, whether attentional problems are frequently associated with mnestic problems, and vice versa. Selective attentional problems were found in 21 individuals, and selective mnestic problems in 20 individuals. In only 3 patients, a combination of these deficits was found.

## Discussion

In a sample of help-seeking individuals the predictive value of an unsupervised, self-administered online instrument was tested. The results revealed that the necessity for an intervention—as indicated by the in-person examination—was signaled by the preceding online instrument with a high specificity and sensitivity. Two indicators, recall performance in a face-name paired association test and discrimination ability in a visual working memory test, served as reliable predictors. In contrast to our hypothesis, self-reported cognitive deficits were not related to the performance in the in-person testing. Furthermore, the sensitivity and specificity of the online instrument were comparable for the prediction of attentive and mnestic problems in help-seeking individuals. Referring to our initial hypotheses, we will discuss these results in detail in the following.

*Hypothesis 1* was related to the prediction of cognitive deficits—irrespective of the cognitive domain. Here, the NOS revealed good specificity (0.75) and sensitivity (0.80)^40^. A corresponding self-administered test (eSAGE, see Charalambous, 2020) revealed a higher specificity (0.90), but a lower sensitivity (0.71) in the differentiation of cognitive impairment (dementia/MCI) and normal cognitive function^41^. Notably, Scharre and colleagues examined older patients (> 50 years) and identified patients with a neurodegenerative disease. This resulted in a higher boundary for deviances and consequently a very good specificity. In contrast, the present study did not focus on neurodegenerative disease but a much broader spectrum of cognitive deficits through a diverse age range. Compared to the eSAGE, the NOS might be more suited to screen for general deficits in older as well as younger adults. At the same time, eSAGE seems more suited for an older population specifically for those at risk of a neurodegenerative disease. All participants concluded the NOS before the in-person testing. Therefore, we cannot rule out carry-over effects such as performance expectation effects. However, such effects seem unlikely due to the circa one-week time lag and apparent difference in test material and modality.

Despite of the good—overall—predictive quality, the specific predictors stated in hypothesis 1 were not confirmed: In contrast to recognition, recall performance was more informative. As for the visual memory task, the maintenance of the form information was more informative than the binding condition. Finally, the level of subjective cognitive complaints did not contribute to the model.

The lack of the predictive value of recognition performance is probably due to the level of difficulty of the face-name association test: A considerable number of participants performed at ceiling (32%). This leads to a reduced variance (SD = 2.23). The larger variance in the recall performance (SD = 3.25) allows a better discrimination of interindividual differences in performance, and therefore serves as a better predictor. Another factor could be that deficits in recognition are more prevalent in patients with neurodegenerative disease^26,27^. Notably, the group of individuals in our sample with cognitive deficits is not significantly older than the group of help-seekers without marked cognitive problems (see supplement), and the cognitive problems are unlikely to signal the onset of a neurodegenerative disease. Notably, the predictive quality of the recall performance rather resembles the everyday concerns of this group of help-seekers, and have a high ecological validity.

The binding performance in the visual short-term memory task did also not serve as a significant predictor. Again, this prediction was based on clinical findings in patients with a neurodegenerative disease^29^: In an Alzheimer’s dementia group, the performance was selectively reduced in the binding, but not in the form-condition. Again, we have to consider that our sample was defined by younger help-seekers (mean age in Della Sala study: 69.35 years vs. 48.22 years in our sample). In this group, the problems in the form-condition can rather be related to the processing and maintenance of unfamiliar visual objects. The task requires a compensatory strategy, such as finding names for the unfamiliar shapes. Importantly, the ability or inability to develop such a strategy itself might contribute to the predictive power of the form-condition. In fact, previous studies show that internal strategy use correlates with working memory performance^42^ and cognitive health^43^. Help-seekers with cognitive problems requiring an intervention might struggle finding an adequate strategy.

Lastly, we supposed that the level of subjective cognitive complaints will be related to the necessity of a neuropsychological intervention as indicated by the in-person testing. This idea was based on a series of previous findings which revealed a relation between the subjective complaints and the objective test performance^16,44–46^. These findings are in line with the idea that SCI could signal a first manifestation of neurocognitive disease^6,47^. However, we have to consider that SCI and objective cognitive performance as measured by gold standard neuropsychological tests are often not strongly associated, despite high prevalence of SCI, especially in older people^5^. Given the younger age in our sample, we suppose that our help-seekers might apply compensatory mechanisms which keep performance at a normative level while causing subjective strain in the ease of cognitive function^48,49^.

Hypothesis 2 was focusing on the predictability of specific cognitive functions, memory and attention. First, we have to consider the interdependence of the domains: Attentional deficits can influence memory deficits and vice versa^50^. However, in our sample the overlap is rather small: Only in 3 patients, a combination of the deficits was observed. In other words: A deficit in the attentive domain does not necessarily affect the mnestic performance.

Given the design of the NOS, its predictive value should be more closely related to the mnestic domain compared to attentive domain. The model parameters partially support this idea: According to the effect size measures, the model predicting memory function appears to be superior to the model for attentional function (Nagelkerke Index, memory = 0.316 > attention = 0.203). The same accounts for the specificity and sensitivity (0.78 and 0.78 for memory > 0.68 and 0.74 for attention).

The significant fit of models for both domains, however, indicate that the predictability of the NOS results is not exclusively valid for the mnestic domain. This extends to the significant predictors included in the regression models. The FNAT delayed recall performance is a significant predictor for both, memory and attentional function deficits. As for the mnestic functions, this result is not surprising as there is a high convergent validity between the classification based on the FNAT and the VLMT/CERAD score (see supplement Table S2). Performance in a delayed recall task is an established parameter for memory function in clinical diagnostics^51,52^. Its relation to attentive function is less clear: Focusing attention is a prerequisite for successful learning, and—correspondingly—a moderate relationship between attention and delayed recall has been reported^53^. According to our data, deficits in selective attention do not primarily affect the acquisition process (reflected in the learning rate), but the subsequent free recall. This preliminary conclusion remains to the confirmed in further studies.

In contrast to the face-name-association test, the short-term memory binding test provides different predictors: The performance in the binding-condition (form-to-color) was a relevant predictor for memory deficits, supporting previous evidence that binding errors are more frequent in neurodegenerative diseases^54^. Recent electrophysiological findings indicated that binding is associated with a more-elaborative encoding and storage processes—particularly in the elderly^55^. In line with those findings, a neuroimaging study showed that hippocampal and associated medial temporal lobe activity during the encoding phase is predictive for successful short-term memory binding^56^. These processes are apparently also required in the association to be build—either between form and color, or between faces and names. In contrast, attention deficits are rather announced by the performance in the form-condition. Here, individuals are rather forced to maintain unfamiliar visual forms in working memory (see Fig. 2). According to our results, this process is challenged when individuals have deficits in maintaining the focus of attention and selecting the relevant information.

*General recommendation on the use of the NOS*: The data available allow us to compare the NOS with the psychometric properties of other web-based tools available. Following the criteria defined in the review of Charalambous^12^, the NOS appears to be competitive in several respects: Like the BrainCheck^57^, the DANA^58^, or the Cogniciti^59^, the NOS provides normative data, and its validity has been demonstrated. As mentioned before, its sensitivity and specificity are not restricted to the classification of neurodegenerative diseases (see above), but extends to the classification of functional deficits. An open issue is the retest reliability: Since a parallel version is not available, test repetition effects cannot be ruled out. On the other hand, the NOS also considers subjective cognitive complaints which become increasingly relevant in neuropsychological diagnostics^60^.

Despite of its psychometric quality, the NOS is not qualified to replace in-person testing: First, the NOS did not include an assessment of attentive functions based on a measurement of reaction times. This is hard to implement reliably into an at-home online test due to different hard- and software capabilities and in-person testing is recommended^13^. Second, the NOS did not consider the full range of executive functions^61^. Notably, deficits in this domain, such as word finding difficulties, are a sensitive marker in specific neurodegenerative diseases^62^. Third, we have to consider that the sensitivity and specificity still signals a considerable number of misses and false alarms. Lastly, while the models show good accuracy, basing the recommendation of treatment solely on a screening instrument does not satisfy quality guidelines. This is a general restriction that applies to most unsupervised online instruments^12^.

Therefore, we like to highlight the use of web-based instrument, such as the NOS, as a pre-cursor of an in-person testing. In a time-saving manner, the results will provide an idea on the expression of subjective and objective cognitive deficits of an individual. Consequently, they allow to focus the anamnesis, and to guide the selection of the psychometric instruments. Most of all, the satisfactory accuracy of the NOS result might allow us to mark help-seeking individuals with a high priority for an in-person testing.

### Limitations

When looking at the current study, despite best efforts we need to consider a few limitations. (1) As described above, the sample consisted of a group of help-seeking individuals characterized by a high level of subjective complaints, and—partially—a higher level of depression. The help-seeking behavior itself reflects a high motivational state and problem-focused behavior^4^. Another limitation is a selection bias in our sample: It is likely that only HS individuals with sufficient digital literacy will follow the invitation to conduct an online screening before visiting the memory clinic. These sample characteristics might limit the generalizability of our results. (2) The STMB test is often conducted with more than two items presented simultaneously^20,63^. Here, only two items were presented. This was done so that a deviance in performance here would give a strong signal towards cognitive impairment. The use of three items however might increase sensitivity and sharpen the usefulness of the STMB test performance as predictors. (3) For the FNAT relatively homogenous faces (e.g. perceived ethnical background) and names (e.g. commonly used in Germany) were chosen in consideration of an older German target group of the neuropsychological consultation hour. This might increase difficulty of the FNAT for patients of a non-German background and hence decrease suitability for these patients. (4) We have to consider that all participants concluded the NOS before the in-person testing. Although test material differed in both settings, we cannot rule out carry-over effects, such as specific expectations concerning the outcome of the ‘in-person’ testing triggered by the preceding NOS. (5) We took a rather conservative approach by setting the cut-off points at percentage ranks of 5. This was done in order to increase specificity. However, this automatically means lower sensitivity. Thus, some patients who would profit from intervention might be missed. This is a general trade-off when designing diagnostic tests and re-emphasizes the need of in-person testing. (6) Necessity for a longitudinal study: Only the repeated measurement will allow us to classify help-seekers reliably, and to estimate whether an intervention has been helpful.

### Conclusion

The NOS is a self-administered online instrument meant to screen for cognitive deficits. The present study confirms the usability of the NOS. Both younger and older adults were able to successfully use the NOS at home and unsupervised. The validity of the results and the normatively extracted cut-off points is supported by the predictability of an individuals need for cognitive intervention procedures.

## Supplementary Information

Supplementary Information.

## Data Availability

All data and a local version of the online test can be retrieved in an Open Science Framework (https://osf.io/4e9v7/).
