# Supplementary material for: Predictive value of an unsupervised web-based assessment of the neuropsychological function
Source: Sci Rep. 2025 Jan 10;15:1645. doi: 10.1038/s41598-025-85614-x (PMC11724069; doi:10.1038/s41598-025-85614-x)
Supplement: Supplementary file 1 — Supplementary Information. [file 41598_2025_85614_MOESM1_ESM.pdf]

Supplement

Contents

1 Comparison between participants with and without a general deficit in in-person testing 2

2 Descriptive data and correlation between FNAT classification score and in-person memory performance... 3

3 Regression models including all considered predictors (before reduction) ….............4

3.1 General Deficit 5

3.2 Attention 6

3.3 Memory 7

3.4 Executive Function 8

1 Comparison between participants with and without a general deficit in in-person testing

Table S1. Participants with a general deficit in the in-person testing did not significantly differ in age, education and GDS from participants without a general deficit. GDS: Geriatric Depression Scale.

	Participants with deficit	Participants without deficit	t-test
age (years)	M = 48.31 (SD = 16.48)	M = 49.16 (SD = 14.58)	T (180) = .332, p = .740
education	< 12 years: N = 13 (27%)	< 12 years: N = 52 (39%)	T (180) = -1.515, p = .133
	12 years+: N = 35 (73%)	12 year+: N = 82 (61%)	
GDS	M = 6.98 (SD = 3.74)	M = 6.84 (SD = 3.50)	T (180) = -.227, p = .821

2 Descriptive data and correlation between FNAT classification score and in-person memory performance

The FNAT and the VLMT/ CERAD are assumed to reflect a similar construct. To test this assumption, we have calculated the correlation coefficient Yules Q for the association between the classification “memory deficit” (in-person testing) and a variable reflecting the FNAT performance. For this FNAT variable, the FNAT results were classified in the same manner as the VLMT/ CERAD results in the in-person testing: If a participant showed a deficit in two or more FNAT variables (learning, delayed recall, recognition), we assigned a “1” (= deficit) in the “FNAT classification”, otherwise we assigned a “0” (= no deficit). There is a strong association (Yules Q = .811) between the classification based on in-person memory tests and on the FNAT (see table 2). This high convergent validity of classification supports the notion that the tests are related to the same construct.

Table S2: Descriptive data and correlation between the classification based on the FNAT performance and the in-person memory test.

		FNAT classification		
		0 (no deficit)	1 (deficit)	sum
memory	0 (no deficit)	138	27	165
	1 (deficit)	8	15	23
sum		146	42	
Yules Q = .811				

3 Regression models including all considered predictors (before reduction)

3.1 General Deficit

Table S3. In a first step, a binary logistic regression analysis for a general deficit in the in-person testing was calculated. The model returned significant. FNAT delayed recall and A'(F) were significant predictors. This was used as basis for the reduced model reported in the results section. significance level: p<.05*, p<.01**, p<.001***

	B	SE	Wald	df	p	Exp(B)	CI Exp(B)
age	-.028	.015	3.464	1	.063	.973	[.945. 1.001]
education	.259	.473	.299	1	.584	1.295	[.513. 3.274]
GDS	-.152	.562	.074	1	.786	.859	[.286. 2.581]
CPI	.187	.498	.141	1	.707	1.205	[.455. 3.197]
FNAT learning	1.138	.597	3.641	1	.056	3.122	[.970. 10.052]
FNAT delayed recall	1.698	.541	9.865	1	.002**	5.462	[1.893. 15.758]
FNAT recognition	.432	.513	.707	1	.400	1.540	[.563. 4.210]
A‘ (F)	1.368	.615	4.952	1	.026*	3.928	[1.177. 13.107]
A‘ (CF)	.618	.834	.549	1	.459	1.855	[.362. 9.510]
constant	-1.293	.865	2.234	1	.135	.275	
Chi² (9) = 59.836. p <.001***							
Nagelkerke Index = .409							

3.2 Attention

Table S4. In a first step, a binary logistic regression analyses for an attention deficit in the in-person testing was calculated. The model returned significant. FNAT delayed recall and A'(F) were significant predictors. This was used as basis for the reduced model reported in the results section. significance level: p<.05*, p<.01**, p<.001***

	B	SE	Wald	df	p	Exp(B)	CI Exp(B)
age	-.022	.017	1.692	1	.193	.978	[.947. 1.011]
education	.224	.547	.167	1	.683	1.251	[.428. 3.656]
GDS	.359	.575	.391	1	.532	1.433	[.464. 4.422]
CPI	.673	.633	1.129	1	.288	1.961	[.566. 6.786]
FNAT learning	.850	.653	1.690	1	.194	2.339	[.650. 8.417]
FNAT delayed recall	1.434	.600	5.712	1	.017*	4.195	[1.294. 13.596]
FNAT recognition	-.290	.608	.228	1	.633	.748	[.227. 2.463]
A‘ (F)	1.488	.659	5.094	1	.024*	4.426	[1.216. 16.110]
A‘ (CF)	-.286	.832	.118	1	.731	.751	[.147. 3.846]
constant	-2.592	.980	6.997	1	.008**	.075	
Chi² (9) = 28.301. p <.001***							
Nagelkerke Index = .250							

3.3 Memory

Table S5. In a first step, a binary logistic regression analyses for a memory deficit in the in-person testing was calculated. The model returned significant. FNAT delayed recall was the only significant predictor. However, A'(CF) was near significance level. This was used as basis for the reduced model reported in the results section. significance level: p<.05*, p<.01**, p<.001***

	B	SE	Wald	df	p	Exp(B)	CI Exp(B)
age	-.016	.019	.771	1	.380	.984	[.949. 1.020]
education	.467	.609	.588	1	.443	1.596	[.483. 5.267]
GDS	-.482	.762	.400	1	.527	.618	[.139. 2.750]
CPI	-.656	.648	1.024	1	.312	.519	[.146. 1.848]
FNAT learning	.518	.695	.554	1	.457	1.678	[.429. 6.559]
FNAT delayed recall	1.711	.721	5.626	1	.018*	5.536	[1.346. 22.769]
FNAT recognition	.921	.639	2.077	1	.150	2.511	[.718. 8.784]
A‘ (F)	.744	.734	1.027	1	.311	2.103	[.499. 8.863]
A‘ (CF)	1.558	.847	3.386	1	.066	4.750	[.903. 24.977]
constant	-.016	.019	.771	1	.380	.984	
Chi² (9) = 41.511. p <.001***							
Nagelkerke Index = .378							

3.4 Executive Function

Table S6. A binary logistic regression analyses for an executive deficit in the in-person testing was calculated. The model returned non-significant. Therefore, the model was not reduced any further. significance level: $p<.05^{*}$, $p<.01^{**}$, $p<.001^{***}$

	B	SE	Wald	df	p	Exp(B)	CI Exp(B)
age	.002	.037	.003	1	.955	1.002	[.931, 1.078]
education	.239	1.272	.035	1	.851	1.270	[.105, 15.349]
GDS	-17.717	5959.416	.000	1	.998	.000	.000
CPI	18.417	4587.088	.000	1	.997	99681111.205	.000
FNAT learning	1.637	1.832	.798	1	.372	5.139	[.142, 186.392]
FNAT delayed recall	-1.317	2.104	.392	1	.531	.268	[.004, 16.570]
FNAT recognition	.543	1.725	.099	1	.753	1.721	[.058, 50.638]
A‘ (F)	-16.924	7211.637	.000	1	.998	.000	.000
A‘ (CF)	-16.619	9372.547	.000	1	.999	.000	.000
constant	-21.719	4587.089	.000	1	.996	.000	
Chi^2 (9) = 8.344, p =.500							
Nagelkerke Index = .230							
